# Child Sexual Abuse in Mexican Women: Type of Experience, Age, Perpetrator, and Disclosure

**DOI:** 10.3390/ijerph18136931

**Published:** 2021-06-28

**Authors:** Pilar Rueda, Marta Ferragut, M. Victoria Cerezo, Margarita Ortiz-Tallo

**Affiliations:** Faculty of Psychology, University of Málaga, 29071 Málaga, Spain; mferragut@uma.es (M.F.); mvcerezo@uma.es (M.V.C.); mortiztallo@uma.es (M.O.-T.)

**Keywords:** child sexual abuse, maltreatment, childhood

## Abstract

Child sexual abuse (CSA) is a type of maltreatment that occurs in practically all countries and social statuses. Due to the taboo and shame that surrounds it, CSA is a problem universally silenced despite the important consequences (both physical and psychological) that it has for the victim and their family. This work aimed to study the correlates of CSA in Mexican women. Our sample comprised 1058 women ranged from 18 to 73 years (*M* = 40.19; *SD* = 10.24). They completed an anonymous online survey including questions about all the different types of abuse, questions about who perpetrated it, at what age it happened, and whether the victim disclosed the abuse. Our results showed that, depending on the type of abuse, from 13.9% to 65.8% of the participants had suffered at least one episode of CSA throughout their childhood. The first episode typically occurred between 6 and 12 years old, with the perpetrator being a male. The youngest women reported higher rates of being shown pornography by a family member, whereas the oldest ones reported higher rates of exhibitionism by a stranger. Only 31.3% of the sample disclosed the abuse, usually to their mother or a peer. Differences among the correlates of the different types of abuse, the age of the victims, and the relationship with the perpetrator are discussed as well as the victims’ feelings of being believed when they disclosed the abuse.

## 1. Introduction

In recent years, research has highlighted the high prevalence of child sexual abuse (CSA) and the need to clarify not only the prevalence but also the nature of this form of maltreatment and its consequences [1,2]. It is a widespread phenomenon across all countries and all socioeconomic levels and is universally silenced [3,4,5,6,7,8,9,10,11,12].

According to the World Health Organization [13], CSA is one of the most common types of trauma during childhood, with a higher rate for females than for males [1,14]. CSA is defined as the “involvement of a child in sexual activity that he or she does not fully comprehend, is unable to give informed consent to, or for which the child is not developmentally prepared and cannot give consent, or that violates the laws or social taboos of society. CSA is evidenced by this activity between a child and an adult or another child who by age or development is in a relationship of responsibility, trust, or power, the activity being intended to gratify or satisfy the needs of the other person” [15]. According to this definition, it can be considered, first, that for an act to be considered abuse, it does not necessarily imply physical contact. Second, any act of a sexual nature between an adult and a minor is CSA because there is a legal age at which a person is considered to be able to give consent and not before. In Mexico, as a federal country where each state has the autonomy to establish its legislation, the legal age of consent for sexual relationships is between 12 and 16 years old [16].

The consequences of CSA can be both direct (insomnia, somatization, anxiety, depression; [9]) or indirect, such as feelings of anger, shame, relational problems, eating disorders, or personality disorders [17,18]. Research has shown that girls who are victims of CSA in childhood are more likely to experience re-victimization during adolescence or adulthood [19] as well as to have a negative self-image [20]. As these sequelae can turn into physical and psychological illnesses; they are an obstacle to the socio-economic development of the country [21,22].

The definition of CSA used by the studies as well as the data collection procedure, are key elements in terms of prevalence data, which can vary widely from one research to another [1,14]. A recent international meta-analysis found a mean prevalence of 24% [1], with large heterogeneity among countries. Thus, for example, while Europe and Asia showed a prevalence of 17% and 18%, respectively, in the USA, the prevalence was as high as 32%. These data should always be interpreted carefully, as cultural values play an important role not only in the experience of abuse but also in the person’s willingness to talk about it. The family’s honor, the culture of individuality, the value of children’s obedience to the adult no matter what, the stigmatization of victims, and different gender roles make disclosure difficult in Chinese and Latin American cultures [2,20], even during adulthood.

In Mexico, Pineda-Lucatero et al. [9] found that 18.7% of adolescents had suffered sexual abuse during their childhood, with the percentage being higher among girls than boys. These authors analyzed different types of abuse and who the perpetrator was, finding that abuse carried out by a family member reached 37.3% in girls and 31.7% in boys. Distinguishing between different forms of abuse and the person who carries it out is a keystone in understanding the phenomenon and its consequences [5,18]. It is a question of both theoretical and practical relevance to know whether there are differences not only in the frequency with which abuse occurs but also in the type of abuse and its consequences on the victims’ mental and physical health.

In the study by Pineda-Lucatero et al. [9], the average age of occurrence of the first episode was 7.5 years. In another interesting study, also carried out within a Mexican population, Quintero [20] interviewed female victims of abuse and pointed out the importance of culture not only in the perception of abuse but also in the self-image that victims create as a result of this event. The importance of knowing the culture’s values and myths about CSA has been highlighted in other studies [23,24].

The impact of trauma on the child and subsequently on the adult differs according to the type of abuse suffered, the duration of the abuse, and who perpetrated it [18,20,25]. Research has shown that, in clinical samples, traumatic symptomatology is higher when the abuser is a person who is not the child’s caregiver. However, when studying non-clinical samples, the opposite result is observed, that is, a greater impact of trauma if the abuse is perpetrated by a caregiver [17].

CSA is a complex phenomenon. Its very nature implies that victims do not always dare to report it out of fear and/or shame, and most of the cases are not reported to the authorities. It is estimated that only one in three victims disclose it while it is happening [26,27], and of these, 40% tell a peer, so it is often not known by the adults who take care of the child [28]. Research has shown that only 10–15% of the cases are reported [29,30]. In Mexico, most of the cases reported by children fail to lead to legal proceedings, remaining in the National System for the Integral Development of the Family [31]. It is estimated that only one in ten cases of abuse is reported in Mexico [32]. This difficulty in disclosure is worsened by the high frequency with which victims later retract their reports due to family pressures, not being believed, or fear of stigma and shame [33,34,35].

These difficulties in learning about the existence of abuse at the time of its occurrence mean that most prevalent studies are conducted retrospectively, that is, by asking adults about their childhood [35]. Such studies have shown that giving participants a clear definition of what is considered abuse is a key determinant of whether or not they identify themselves as victims of abuse [29,35].

This study aimed to know the frequency of occurrence of different types of CSA in a sample of adult Mexican women. The questions were asked retrospectively, asking participants about their childhood and adolescence via an online questionnaire and focusing on six different types of sexually abusive experiences. We also explored at what age the first episode occurred, who carried out the abuse, whether the victim told anyone as a child, and whether they felt that this person believed them. Finally, the number of different perpetrators in relation to the age of the victim at the time of the first episode was analyzed.

## 2. Materials and Methods

### 2.1. Participants

Participants were 1058 Mexican women between 18 and 73 years old (*M* = 40.19, *SD* = 10.24). Their level of studies were primary, *n* = 60 (5.7%); secondary or non-university studies, *n* = 164 (15.5%); and graduates or studying at University at the time of the study, *n* = 834 (78.8%). To participate in this study, they had to be be older than 18 years old and had to sign the informed consent.

### 2.2. Instrument

**Child Sexual Abuse Questionnaire (CSAQ)**. This questionnaire was developed by the research team and published in an earlier study [5]. It collects demographic information about gender, age, educational level, and nationality and information related to six types of child sexual abuse experiences. This information altogether provides a detailed description of the sexual experiences of the participants during their childhood and adolescence. Participants were asked to focus on abusive experiences that occurred (1) when they were still younger than 18 years old, (2) with an adult or with a child at least five years their senior, and (3) which caused them discomfort or distress. The general question, which considered six events, was whether an adult or an older child had ever (yes, no): (1) rubbed their private parts against you, (2) fondled any part of your body, (3) touched your private parts, (4) showed you pornographic material, (5) showed you his/her genitals, or (6) asked you to touch their private parts. If a participant answered yes for any of these six experiences, she was then asked to specify her age at the time of the first episode (response options were: younger than six; between 6 and 12; 13 to 18; I’m not sure/don’t know) and to indicate the perpetrator’s gender and her relationship to them (family member/relative; teacher, instructor, baby-sitter, etc.; acquaintance, such as a friend or neighbor, etc.; or a stranger). Participants could indicate more than one option. Finally, participants were asked to state whether they had talked to anyone about these experiences while still a minor (yes, no) and, if so, whom they had talked to (mother; father; a schoolmate or peer of the same age; an adult relative; a teacher or instructor) and whether this person had believed them (yes, no).

### 2.3. Procedure

The study was carried out in accordance with the Declaration of Helsinki and was approved by the Research Ethics Committee of the University. Participants were informed about the aims of the study, and then a snowball sampling strategy to recruit participants was used. All of the participants were asked to sign a declaration confirming that they were over 18 years of age, that they had been informed about the purpose of the study, and that they agreed to participate voluntarily and anonymously before accessing the survey. The survey was available online for six months (January–June 2019), and was sent via e-mail and posted through social media platforms. It took participants around 10 min on average to complete.

### 2.4. Data Analysis

To analyze the prevalence of CSA in this group, percentages for each of the six types of experiences were computed. To analyze the context and variables of these CSA experiences, we also computed:

- The number of different sexual abuse types suffered by adding “yes” responses to the six types of experiences and classifying three categories: 1, 2-3, 4 or more;
- The type of contact experienced, categorizing the sexual abuse into “with physical contact” (a “yes” response for being rubbed, fondled, touched, or asked to touch the perpetrator), “without physical contact” (a “yes” response for being shown the perpetrator’s genitals and/or pornography), or both.
- The number of different abusers by adding “yes” responses to different relationships with the abuser and classifying into: 1, 2, or more;
- The victim’s age when the first episode of sexual abuse occurred: younger than six years old, between 6 and 12 years old, and between 13 and 18 years old.
- The gender of the abuser and the relationship with them, classified into: family or relative, stranger, acquaintance, teacher or instructor.
- Variables related to the disclosure: whether the victims reported the incident to somebody else, to whom, and whether they were believed.
- The relation between the age of the victim at the time of the first episode and the variables of the number of CSA types, the type of contact experienced, and the number of abusers were analyzed using the chi-square test.

The questionnaire was designed with key mandatory questions, so incomplete questionnaires could not be submitted, preventing missing data from the study. Nevertheless, in those cases where the participant did not answer a non-mandatory question (for example, after responding “yes” to have suffered any type of abuse, they were directed to a question about who perpetrated it, and some participants did not answer), these questions were computed as “non stated”.

## 3. Results

The prevalence of CSA reported by this group of Mexican women ranged between 13.9% and 65.8% depending on the CSA type, most commonly being rubbed against or fondled. The less prevalent type of abuse was being shown pornographic material (see Table 1). The percentage of women who claim to have suffered some form of sexual abuse during childhood and adolescence represents 77.69% of our sample (Table 1).

A total of 822 women (out of 1058) reported having suffered at least one of the experiences while being under 18 years old. Of those women, 18. 2% reported suffering one experience, 40.8% suffered two or three experiences in their lifetime, 41% suffered 4 or more different types of experiences.

Regarding whether there was physical contact, only 3.8% reported that the episode occurred without physical contact, 44% reported physical contact, and 52% reported both kinds of experiences, with and without contact.

Focusing on the different number of perpetrators who had abused them, half of the victims (50.1%) had been abused by one perpetrator, 29.1% reported they had been abused by two or more perpetrators, and 20.8% of the sample did not answer this question.

Table 2 shows the age at the time of the first episode, the relationship with the perpetrator, and their gender. It also shows whether the girl told someone about the abuse and if so, whom she told, and finally, whether that person believed her.

The results show that the first episode occurred mostly between 6 and 12 years old (47.9%), followed by being younger than 6 years old (26.6%), and between 13 and 18 years old (18.1%).

Concerning the perpetrator, 48.1% responded that it was a family member or a relative, and 43.8% said that he was a stranger, and 30.9% that he was an acquaintance. Only 11.6% claimed to have been abused by a teacher. The gender of the perpetrator was mostly male (79.7%), but 2.1% reported being abused by a woman.

Concerning the question of telling someone about the abuse, only 29.3% talked to someone, and 63.7% never talked to anyone about it during their childhood. When the girl told someone, the mother was the most frequently chosen person (66.8%), someone of the same age in second place (21.2%), a relative in third place (10.4%), and finally, the father (1.2%) or a teacher (0.4%). The majority of women who talked about it responded that they were believed (77.6%) (Table 2).

Table 3 analyzes the quantity of the different types of sexual abuse (with and without physical contact) and the number of different perpetrators in relation to the age of the victim at the first time of the episode. Results show that being younger than six years old at the first episode was significantly related to suffering more experiences and different types of abuse, with both contact and non-contact. In contrast, victims older than 13 years old at the time of the first episode reported higher rates of only one perpetrator.

## 4. Discussion

The objective of this study was to investigate the frequency of occurrence of different types of CSA in a sample of Mexican women. We analyzed six different types of abusive experiences during childhood and adolescence. We also explored who perpetrated the abuse, at what age the first episode occurred, how long it lasted, and whether still as children and adolescents they told someone and were believed. Finally, we analyzed whether there was physical contact and the number of different abusers in relation to the victim’s age at the time of the first episode.

The prevalence of different types of CSA ranged from 13.9% to 65.8%, and 41% suffered four or more different types of the experience in their lifetime, 52% reported experiences with and without contact. Depending on the type of abusive experience, the percentage varies; being rubbed against or fondled was the most frequent (65.8% and 58.5%). The less prevalent type of abuse in this group of Mexican women was being shown pornographic material (13.9%). These results are higher than those obtained in the meta-analysis of prevalence among women using the *Childhood Trauma Questionnaire* [1]. The results reported by Ferragut et al. [5] in Spanish women using the same questionnaire as the *Child Sexual Abuse Questionnaire* (CSAQ) ranged between 9.8% to 53.1%, lower than those obtained in this study. Nevertheless, this Spanish study also showed being rubbed against as the most prevalent type of abuse (53.1%) and the perpetrator showing pornographic material to the child the least frequent (9.8%).

Studies on the prevalence of CSA worldwide have shown that this phenomenon is not exclusive to some countries and is spread across all socioeconomic levels. Guziack [36], through an online questionnaire in Poland, found a CSA prevalence of 46.7% in women. The prevalence found in different countries ranges from 17% in Europe and Asia to 32% in the USA [1]. Latin American literature has reported a prevalence of 13.4% in women [37]. In Mexico, this prevalence was estimated at 13.3% using the National Survey of Violence against Women (ENVIM; 2003; [38]) but increases to 18.7% when asking adolescents through a survey about CSA developed for the study [9].

The gender of the abuser was mostly male (79.7%), in line with previous studies, although 2.1% responded that they had been abused by a woman. Usually, the perpetrator was known by the victim—a family member or an acquaintance—but also a high percentage was unknown to the victim. These results are similar to those found in other studies [5,25,39].

The first episode of sexual abuse often occurred between the age of 6 and 12 in this Mexican sample. This seems to be the age with the highest risk of CSA, as other studies in Mexico have reported [9] and is also consistent with results found in previous studies carried out in other countries [5,40].

In this study, being younger than six years old at the first episode was significantly related to suffering more experiences and different types of abuse with both contact and non-contact. Victims older than 13 years reported higher rates of being abused only by one perpetrator, and these results are in line with the scientific literature [5,19,25].

The relation between the victim’s age and the number of episodes is disturbing because the younger the victim, the more likely she is to suffer four episodes or more and also more likely to have different abusers. The age also determines the existence of physical contact; the younger the victim (younger than six), the more likely to suffer abuse with and without physical contact, whereas the older victims more commonly suffered abuse with physical contact [5,25,41].

Different investigations found that suffering CSA leaves important scars, more serious ones depending on the type of abuse, its duration, the number of perpetrators, and the age of the victim [18,19,20,25].

The majority of victims wait until adulthood to reveal their abuse experience [27,42]. Clinicians working with adults are used to discovering during therapy that the patient or client was a victim of CSA even when this was not the reason for therapy. Many of them are reluctant to disclose their experiences due to several factors, such as the culture’s values (family honor, children seen as forced to be always obedient to adults), the stigma of shame, promiscuity or homosexuality, fear of not being believed, and fear of the consequences or not understanding what is happening to them [2,20].

In this sense, the results show that only 29.3% of women reported their experience to someone as girls. London et al. [26] and Ullman [27] found similar results in the USA. Ferragut et al. [5] found higher percentages of disclosure in Spain. In the present study, we found that more than half of the victims who reported being abused told their mother, followed by telling a relative and a same-age friend.

To design programs and policies to protect children against this maltreatment is crucial for a healthy and productive society [21]. However, this is a difficult task because CSA continues to be concealed and difficult to talk about.

Our results reveal higher percentages than those reported in the literature but, far from triggering fear and alarm, the objective of this study was to shed some light on the experiences with CSA of a large group of Mexican women. We cannot extend these findings to the whole Mexican population, but they must be taken into account as a piece of the reality of CSA in this country.

We note as a limitation of the studies on this subject that the definition of CSA and the instrument used can bias the results obtained [1,14,25]. The instrument used in this study, which comprehends different types of abuse with and without physical contact, the perpetrator or perpetrators, the number of episodes, and whether the victim dared to report it and was believed overcomes at least some of the limitations mentioned, as it provides a broader view of Mexican women’s experiences with CSA. Despite this, recruiting participants online has advantages and disadvantages, for example, we have to trust that all of the participants were actually women. Also, since not all of the population have internet access, the sample could be skewed. In fact, a high percentage of our participants had university studies. All in all, and being aware that this sample is not representative of the whole Mexican population, 77.69% of women reporting at least one experience of abuse was shocking and reinforces the idea that more policies and programs to prevent CSA are needed [43]. The most common type of abuse was “being rubbed against by the perpetrator” and “being fondled in any part of the body”. Most of the participants who reported abuse suffered different types of abuse, and the majority of them described this abuse as implying physical contact, as observed in the previous section. These numbers prove the high frequency with which CSA occurs even though it is concealed, especially when we find that more than half of the participants who reported being abused during their childhood also reported that they did not tell anyone while still a child. This “silence” is present in all countries [10,12], and Mexico is no exception [32,43]. This silence is a great obstacle for society to gain awareness of CSA and makes us wonder what can we do better as parents, educators, and active members of society so that, if a child is suffering this type of maltreatment, she will feel capable of seeking help. Of the participants in our study who talked to someone, the majority choose their mother and, fortunately, most of them were believed. It is known that to seek help from an adult who the child trusts and not being believed or listened to worsens the trauma [20].

The results presented also confirm the results of the literature about the perpetrator’s gender (79.7% were male) but bring up another perspective about the relationship between the child and the perpetrator. The studies usually show that CSA occurs more frequently within the family context. However, when we consider that being rubbed against is a type of abuse, females are more likely to become a victim, for example, in public transport or a queue. A broad vision of the definition of CSA is crucial to really know how common it is and the need for sexual education so children can protect themselves and adults can understand that a child’s body belongs to them; it is not the adult’s property [21].

Our results cannot be considered a prevalence but a correlate of experiences in a large group of Mexican women, and the data presented are very high in comparison with the prevalence estimated in previous studies [1,9]. Several reasons can explain these differences. First, the instrument used covers types of abuse that do not imply physical contact, such as being shown pornography and acts that can be considered unintentional, such as rubbing the body against a child’s body. Second, it includes a clear definition of what constitutes CSA, specifying that “the situations presented refer to acts that occurred with an adult or someone at least five years older than you or with authority/power over you”. Third, the anonymous procedure of responses and the possibility of responding in a safe environment such as the home can help the women to feel comfortable when being honest. Also, as usually occurs in this type of study, another variable to take into account is that women are more engaged and willing to talk about CSA than men [23]. Several hypotheses have been proposed for this difference, for example, the fact that CSA occurs more frequently to girls or the influence of socio-cultural and educational patterns [23]. It will be of interest for future studies to make cross-national comparisons using the CSAQ.

As has been shown, the better we define the problem, the better we can approach and dig deeper into the reality underlying CSA. It is crucial for society as a whole to understand the importance of educating children to protect themselves by teaching sexual education in accordance with their age and to promote policies and social programs to inform and educate adults. Some cultural values, such as considering children as objects or as adults’ property, need to be changed to provide children with the safety they need to feel. At the same time, adults should be prepared to detect a child at risk of being abused and listen to them and believe them so that the victims are always believed and receive the help they need to grow up healthy and supported.

## 5. Conclusions

Nowadays, CSA remains a worldwide problem that should be addressed from different perspectives: policies, justice, education, and the health system. It is important to gain a better understanding of this phenomenon in order to provide help to the victims and to prevent its occurrence. This study, comprising a great group of Mexican women (*n* = 1058) aimed to know whether they have suffered CSA, the type of abuse, the age they were when the first episode occurred, who was the abuser, and if they told someone as a child or adolescent and they were believed. The results showed that a great percentage of the sample **(77.7%) had been victim of some type of CSA**, the most frequent being to be rubbed against by perpetrator. Most of the participants had suffered four or more types of abuse with both physical and non-physical contact. Regarding the age of the first episode, **it occurred more frequently between 6 and 12 years**. We found than the younger the victim at the time of the first episode (being less than 6 years old), the more episodes of abuse they suffered, and those were carried out by different abusers. Almost half of the sample reported being abused by a relative, this abuser being a man. Also, more than half of the participants did not tell anyone about the abusive situation while they were a child or an adolescent, and among those who did, the majority told their mother and were believed.

These results, although they cannot be considered as a prevalence, highlight the urgent need of protecting children against CSA by teaching them when a “game” or behavior is abusive and to seek help from an adult and by teaching adults (families and educators) to recognize that something could be happening to their child and how to help them.

## Figures and Tables

**Table 1:** Number of participants and percentages for the variables of sexual abuse studied.

Variables of CSA Studied	*n* ^1^	%
Total of women reporting CSA	822	77.7
Types of sexual abuse (yes)		
Rubbed against by perpetrator	696	65.8
Fondled any part of victim’s body	619	58.5
Touched victim’s private parts	455	43.0
Perpetrator showed his/her genitals	423	40.0
Was asked to touch the perpetrator’s private parts	248	23.4
Shown pornographic material by perpetrator	147	13.9
Number of different sexual abuse types suffered (*n* = 822)		
1	150	18.2
2 or 3	335	40.8
4 or more	337	41.0
Type of contact (*n* = 822)		
Without physical contact	31	3.8
With physical contact	362	44.0
Both	429	52.2
Number of different perpetrators (*n* = 822)		
1	417	50.1
2 or more	234	29.1
Not stated	171	20.8

^1^ The group was *n* = 1058.

**Table 2:** Number of participants reporting CSA and percentages for the variables according to age, relationship to the perpetrator, who they told, and whether they were believed.

Variables	*n* = 822	% 77.6
Age at time of first episode		
<6	219	26.6
6–12	394	47.9
13–18	149	18.1
Not stated	60	7.3
Relationship with the perpetrator		
Family/relative	395	48.1
Stranger	360	43.8
Acquaintance	254	30.9
Teacher, instructor, etc.	95	11.6
Perpetrator’s gender		
Male	655	79.7
Female	17	2.1
Not stated	150	18.2
Talked to someone		
Yes	241	29.3
No	524	63.7
Not stated	57	6.9
Who did you talk to? (*n* = 241)		
Mother	161	66.8
Schoolmate or peer (same age)	51	21.2
Relative	25	10.4
Father	3	1.2
Teacher or instructor	1	0.4
Did they believe you? (*n* = 241)		
Yes	187	77.6
No	53	22.0
Not stated	1	0.4

**Table 3:** Percentage of the number of episodes of sexual abuse, their types, and the number of perpetrators according to victim’s age at the first episode.

	Age at Time of the First Episode			
Variables	<6	6–12	13–18	*χ^2^*
Number of sexual abuse types				
1	4.6	11.4	33.6	122.78 ***
2-3	29.7	45.9	51.7	
4 or more	65.8	42.6	14.8	
Physical contact (yes/no)				
No physical contact	1.4	4.1	6.0	35.42 ***
With physical contact	31.1	40.1	57.0	
Both	67.6	55.8	36.9	
Number of perpetrators				
1	59.0	59.1	82.0	23.87 **
2 or more	41.0	40.9	20.7	

** *p* < 0.01; *** *p* < 0.001.

## Data Availability

The data presented in this study are available on request from the corresponding author.
